# Serum ACSL4 levels in prostate cancer patients and its relationship between patient prognosis: A prospective observational study

**DOI:** 10.1097/MD.0000000000039119

**Published:** 2024-10-04

**Authors:** Hao Wang, Hanfeng Xu, Yuan Yang

**Affiliations:** a The First Affiliated Hospital, Hengyang Medical School, University of South China, Hengyang City, Hunan Province, P. R China.

**Keywords:** ACSL4, early diagnosis, prognosis, prostate cancer, PSA

## Abstract

In this prospective observational study, our objective was to investigate the serum levels of Acyl-CoA synthetase long-chain family member 4 (ACSL4) in prostate cancer (PCa) patients and examine its association with other serum biomarkers, and the clinical outcomes of PCa patients. This prospective observational study was conducted from January 2019 to October 2021, including 103 cases of PCa patients and 101 cases of benign prostate hyperplasia (BPH) patients who received treatment at our hospital. All patients had their serum ACSL4 levels measured using enzyme-linked immunosorbent assay before treatment. The clinical outcomes included age, body mass index, gender, systolic blood pressure, diastolic blood pressure, tumor node metastasis stage, Gleason scores, and prostate volume and serum biomarkers were collected. All patients were followed up for 36 months, the overall survival and disease-free survival were recorded for all patients. All data used SPSS 26.0 for analysis. The phosphorus (P) and serum low-density lipoprotein cholesterol levels were significantly higher in PCa patients compared to BPH patients. Furthermore, compared to the BPH patients, the serum ACSL4 and free prostate-specific antigen levels were significantly decreased while serum total prostate-specific antigen (tPSA) levels were significantly elevated in PCa patients. Pearson correlation analysis showed a positive correlation between ACSL4 levels and free prostate-specific antigen levels, while a negative correlation was observed with P and tPSA levels. ACSL4 might serve as a biomarker for diagnosing PCa with the AUC was 0.747, cutoff value of 33.68 ng/mL, sensitivity of 70.3%, and specificity of 60.2%. Finally, we found that ACSL4, tPSA, and P were identified as risk factors associated with PCa patients. Our findings indicated that the serum levels of ACSL4 were significantly decreased in PCa patients compared to BPH patients. Serum ACSL4 could be used as a potential biomarker for early PCa diagnosis and prognosis.

## 1. Introduction

According to Global Cancer Statistics 2020, prostate cancer (PCa) is the most commonly diagnosed male cancer in 112 countries, and it ranks second in terms of mortality among all types of cancer in male cancer patients, second only to lung cancer.^[1,2]^ Due to population growth and aging, it is projected that by 2040, global PCa will increase to nearly 2.3 million new cases and 740,000 deaths.^[3]^ Early-stage PCa often presents no apparent symptoms, while late-stage PCa has poor overall treatment efficacy and high mortality rates. Despite the emergence of various new technologies and endocrine therapies for late-stage PCa, the mortality rate of PCa continues to rise.^[4]^ Therefore, although prostate-specific antigen (PSA) levels have been widely used in the diagnosis of PCa,^[5,6]^ there is still a need to identify new serum markers for early detection of PCa, which would significantly improve patient treatment and prognosis.

Acyl-CoA synthetase long-chain family member 4 (ACSL4) is a key enzyme involved in regulating lipid composition and is involved in ferroptosis, a form of regulated cell death.^[7,8]^ In cancer, ACSL4 acts as a tumor suppressor gene and promotes ferroptosis in cancer cells.^[9,10]^ In a recent review by Wang et al, it was demonstrated that induction of ferroptosis or modulation of ferroptosis-related genes could inhibit prostate tumor growth.^[11]^ Additionally, in vivo regulation of ACSL4 to induce ferroptosis was found to block the growth and metastasis of RB1-deficient prostate tumors.^[12]^ Furthermore, low expression of ACSL4 has been observed in PCa patients according to the cancer genome atlas database (GEPIA, http://gepia.cancer-pku.cn/index.html). These findings suggested that ACSL4 might suppress the development of PCa through regulation of the ferroptosis pathway. However, there is currently a lack of clinical studies investigating the serum levels of ACSL4 in PCa patients and its association with the prognosis of PCa patients.

In this prospective observational study, our objective was to investigate the serum levels of ACSL4 in PCa patients and examine its association with other serum biomarkers, and the clinical outcomes of PCa patients. This study could elucidate the clinical relevance of ACSL4 in PCa patients, and also present novel avenues for future research in PCa treatment.

## 2. Methods

### 2.1. Participants

This prospective observational study was conducted from January 2019 to October 2021, including 103 cases of PCa patients and 101 cases of benign prostate hyperplasia (BPH) patients who received treatment at our hospital. All patients underwent biopsy sampling and histopathological examination, leading to a confirmed diagnosis of PCa or BPH. Exclusion criteria included: (1) patients who underwent radiotherapy, chemotherapy, or immunotherapy before surgery; (2) patients with tumor node metastasis stage III–IV or aged over 70 years; (3) patients with congenital immune deficiency, diabetes, or hyperthyroidism; (4) patients with severe infections, hepatic or renal dysfunction, or pulmonary diseases prior to surgery; (5) patients with other concomitant malignancies. All patients underwent radical prostatectomy performed by the same surgical team at our hospital, and no residual tumors or metastatic lesions were found postoperatively. This study has obtained ethical approval from the Ethics Committee of our Hospital. All eligible participants voluntarily agreed to participate in this study and signed an informed consent form.

### 2.2 . Blood sample measurement

All patients had their serum ACSL4 levels measured using enzyme-linked immunosorbent assay before treatment. Fasting antebrachial vein blood samples (5 mL) were collected from all study participants. After centrifugation at 2000 *g* for 15 minutes, the samples were analyzed according to the instructions provided by the commercially available assay kit (MBS9331516, MyBioSource, USA).

### 2.3. Observation indicators

The observational variables included age, body mass index, systolic blood pressure, diastolic blood pressure, tumor node metastasis stage, Gleason scores, and prostate volume for all study participants. In addition, we performed complete blood cell analysis using a fully automated biochemical analyzer (Hitachi 7600, Hitachi Corporation, Japan) to record levels of low-density lipoprotein cholesterol (LDLC), high-density lipoprotein cholesterol (HDLC), triglycerides (TG), total cholesterol (TC), serum phosphorus (P), serum calcium (Ca), alkaline phosphatase (ALP), total prostate-specific antigen (tPSA), free prostate-specific antigen (fPSA), and free-to-total prostate-specific antigen ratio. All patients were followed up for 24 months, during which recurrence or death was defined as an adverse prognosis. Overall survival and disease-free survival were recorded for all patients.

### 2.4 . Statistical analysis

All data used SPSS 26.0 for analysis. The comparison between 2 groups with a normal distribution was conducted using the Mann–Whitney test. For the comparison between 2 groups with a non-normal distribution, the Student *t* test was employed. The Chi-square test was used for comparing ratios. Spearman rank correlation was used for correlation analysis. The role of serum ACSL4 in the diagnosis of PCa patients was analyzed using ROC curve analysis. Kaplan–Meier (K–M) curve analysis was utilized to compare overall survival and disease-free survival among PCa patients. Multivariable logistic regression analysis was performed to identify risk factors of PCa patients. *P* < .05 regarded a significant difference.

## 3. Results

### 3.1 . Clinical characteristics of all participants

**Figure 1.**
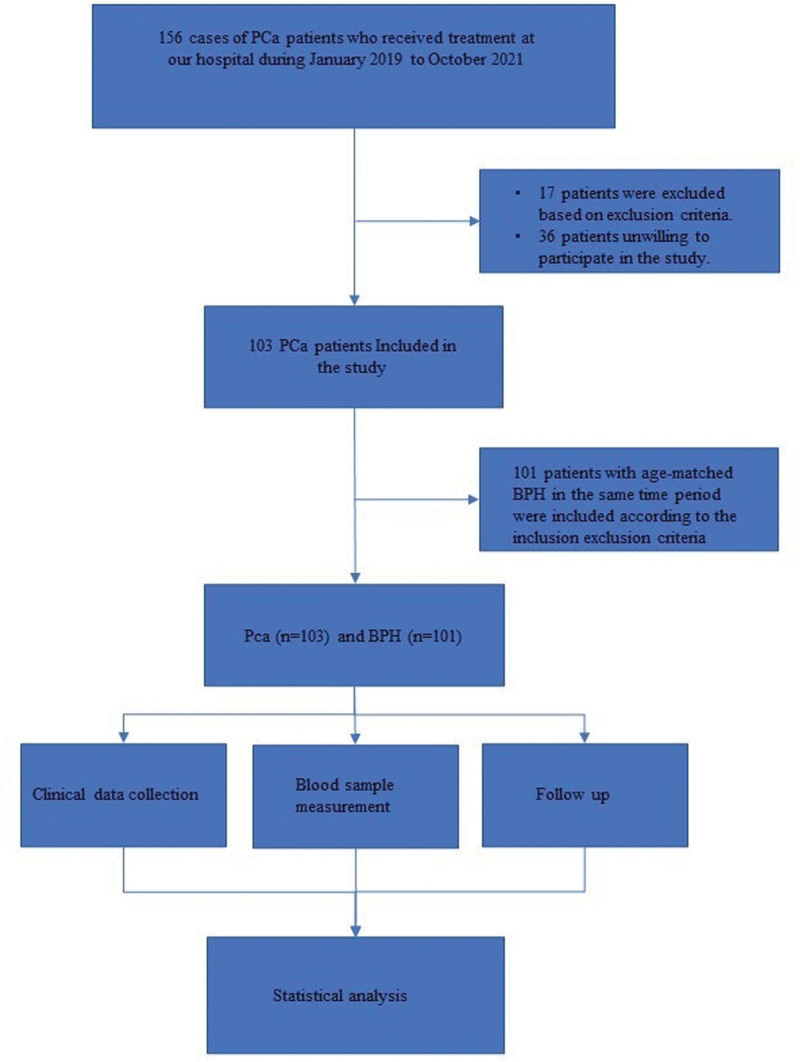
Flow diagram of the study.

This prospective observational study included 103 patients with PCa and 101 patients with BPH who were treated at our hospital, the flow diagram was shown in Figure 1. When comparing the demographic, clinical, and metabolic parameters between the 2 groups, we found that serum P and serum LDLC levels were significantly higher in PCa patients compared to BPH patients (Table 1, *P* < .05). There were no significant differences between the 2 groups in terms of age, gender, body mass index, systolic blood pressure, diastolic blood pressure, HDLC, TC, TG, Ca, ALP, and prostate volume.

**Table 1:** Demographic and clinical data of all subjects.

Variable	PCa, n = 103	BPH, n = 101	*P*
Age, y	59 (51–69)	60 (49–69)	.739
BMI	26.06 ± 3.28	26.25 ± 3.18	.665
SBP (mm Hg)	132.49 ± 14.25	131.17 ± 14.56	.513
DBP (mm Hg)	90 (67–105)	88 (67–105)	.375
TNM stage			
I	44 (42.7)		
II	59 (57.3)		
Gleason scores	6.72 ± 1.19		
PV (cm^3^)	36.24 (15.40–52.80)	34.62 (15.41–52.97)	.818
P (mmol/L)	1.14 ± 0.11	0.96 ± 0.11	<.001
Ca (mmol/L)	2.17 ± 0.10	2.17 ± 0.12	.871
TC (mmol/L)	3.68 ± 0.59	3.67 ± 0.58	.898
TG (mmol/L)	1.40 (1.02–1.63)	1.35 (1.40–1.62)	.578
HDLC (mmol/L)	1.12 (0.94–1.26)	1.11 (0.93–1.26)	.330
LDLC (mmol/L)	2.75 (1.88–3.40)	2.55 (1.91–3.05)	.003
ALP (U/L)	64.36 (33.93–82.750	61.21 (33.61–84.03)	.440

ALP = alkaline phosphatase, BMI = body mass index, Ca = calcium, DBP = diastolic blood pressure, HDLC = high-density lipoprotein cholesterol, LDLC = low-density lipoprotein cholesterol, P = phosphorus, PV = prostate volume, SBP = systolic blood pressure, TC = total cholesterol, TG = triglycerides, TNM = tumor node metastasis.

### 3.2 . Serum levels of ACSL4 and PSA in PCa patients

**Figure 2.**
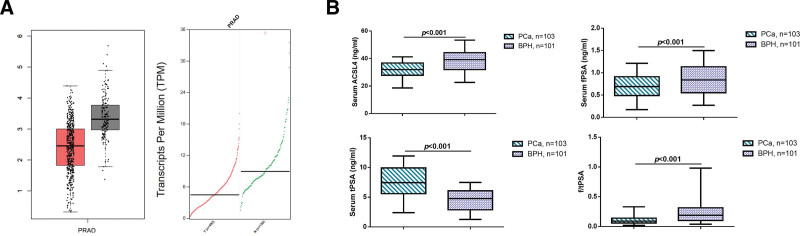
Serum levels of ACSL4 and PSA in PCa and BPH patients. (A) Expression of ACSL4 in PCa patients in the cancer genome atlas (TCGA) database, the red bar represents the expression levels of ACSL4 in prostate adenocarcinoma (PRAD) samples, while the gray bar represents the expression levels in normal prostate tissue samples. (B) Serum levels of ACSL4 and PSA in PCa and BPH patients measured by enzyme-linked immunosorbent assay (ELISA).

The cancer genome atlas database shows low expression of ACSL4 in PCa (Fig. 2A). Further, we measured the serum levels of ACSL4 in all patients using enzyme-linked immunosorbent assay. The results showed that compared to the BPH patients, the serum ACSL4 levels were significantly decreased in PCa patients (Fig. 2B, *P* < .05). Moreover, compared to BPH patients, PCa patients had significantly elevated levels of tPSA and significantly decreased levels of fPSA (*P* < .05).

### 3.3 . Correlation between serum ACSL4 levels and clinical outcome in PCa and BPH patients

Subsequently, we conducted Spearman correlation analysis to investigate the relationship between ACSL4 levels and serum markers in patients. As shown in Table 2, there was no significant correlation between ACSL4 levels and Ca, TC, TG, HDLC, LDLC, and ALP. However, we found a positive correlation between ACSL4 levels and free-to-total prostate-specific antigen ratio (*P* < .05), while a negative correlation was observed with P and tPSA levels (*P* < .05). These results suggest that ACSL4 may be associated with clinical outcomes in patients.

**Table 2:** Correlation between serum ACSL4 levels and the clinical data of PCa patients and BPH patients.

Variable	ACSL4	
	Spearman correlation	*P*
P	−0.304	<.001
Ca	0.123	.080
TC	0.014	.848
TG	0.020	.777
HDLC	−0.054	.443
LDLC	−0.126	.073
ALP	0.096	.173
tPSA	−0.158	.024
fPSA	0.099	.159
f/tPSA	0.191	.006

ACSL4Acyl-CoA = synthetase long-chain family member 4, ALP = alkaline phosphatase, Ca = calcium, f/tPSA = free-to-total prostate-specific antigen ratio, fPSA = free prostate-specific antigen, HDLC = high-density lipoprotein cholesterol, LDLC = low-density lipoprotein cholesterol, P = phosphorus, TC = total cholesterol, TG = triglycerides, tPSA = total prostate-specific antigen.

### 3.4 . Diagnostic value of ACSL4 in PCa patients

**Figure 3.**
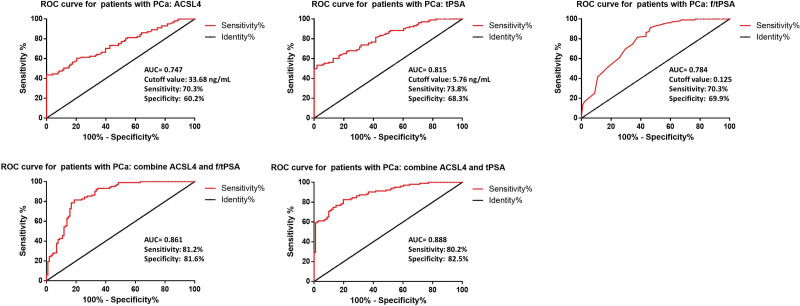
ROC curves in PCA patients diagnosed by serum biomarkers.

To evaluate the diagnostic value of serum ACSL4 in PCa patients, we constructed a ROC curve. The results indicated that ACSL4 may serve as a biomarker for diagnosing PCa. The AUC for ACSL4 in diagnosing PCa patients was 0.747, with a cutoff value of 33.68 ng/mL, sensitivity of 70.3%, and specificity of 60.2% (Fig. 3). In addition, combined ACSL4 and tPSA showed better diagnostic results with an AUC of 0.888, sensitivity of 80.2%, and specificity of 82.5%.

### 3.5 . K–M curve analysis of serum ACSL4 in PCa patients

**Figure 4.**
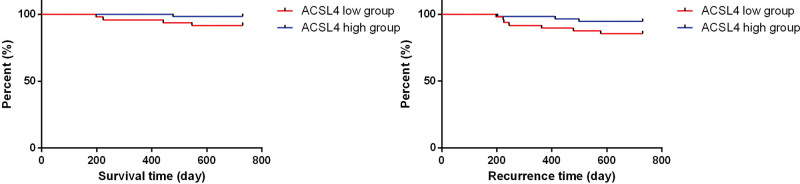
Kaplan–Meier (K–M) curves for two-year survival and recurrence time for PCa patients with high/low serum levels of ACSL4 (the data is not statistically significant).

Then, based on the average serum ACSL4 level of PCa patients (average value 30.60 ng/mL), we divided all PCa patients into 2 groups: ACSL4 high-levels group and low-levels group. We used the K–M curve analysis to study the survival rate and recurrence rate of PCa patients over 24 months. Among all the PCa patients, 5 patients died during the follow-up period, with 2 patients experiencing disease progression and 3 patients experiencing recurrence. The results showed that the low ACSL4 level group had a lower two-year survival rate and a higher recurrence rate, however the data were not statistically significant (Fig. 4).

### 3.6 . Identification of risk factors for PCa patients using logistic regression analysis

To determine the risk factors for PCa patients, we performed logistic regression analysis using the entry method. Our findings demonstrated that ACSL4, tPSA, and P were identified as risk factors associated with PCa patients (Table 3).

**Table 3:** Logistic regression of risk factors for death in IPAH patients.

Variables	Wald	Odds ratio	95% CI	*P*
Age	0.192	1.021	0.929–1.122	.662
BMI	3.587	0.837	0.697–1.006	.058
SBP	0.902	0.983	0.948–1.019	.342
DBP	1.049	1.027	0.976–1.081	.306
PV	1.132	1.027	0.978–1.078	.287
P	24.103	0.001	<0.001–0.001	<.001
Ca	0.212	0.291	0.002–55.942	.645
TC	0.001	0.990	0.380–2.580	.984
TG	0.156	0.467	0.011–20.581	.693
HDLC	0.691	11.675	0.036–3834.329	.406
LDLC	1.914	0.356	0.082–1.538	.167
ALP	0.822	0.982	0.945–1.021	.365
ACSL4	15.351	1.232	1.110–1.368	<.001
tPSA	9.681	0.415	0.238–0.722	.002
fPSA	0.619	3.322	0.167–66.138	.431
f/tPSA	0.009	1.818	<0.001–464744.390	.925

ACSL4Acyl-CoA = synthetase long-chain family member 4, ALP = alkaline phosphatase, BMI = body mass index, Ca = calcium, DBP = diastolic blood pressure, f/tPSA = free-to-total prostate-specific antigen ratio, fPSA = free prostate-specific antigen, HDLC = high-density lipoprotein cholesterol, LDLC = low-density lipoprotein cholesterol, P = phosphorus, PV = prostate volume, SBP = systolic blood pressure, TC = total cholesterol, TG = triglycerides, TNM = tumor node metastasis, tPSA = total prostate-specific antigen.

## 4. Discussion

Although PSA is widely used for PCa diagnosis, its role in asymptomatic PCa screening remains controversial. Research suggests that as a PCa screening test, PSA has limited specificity, leading to overdiagnosis and subsequent overtreatment.^[13]^ Another meta-analysis found that PSA screening increased the diagnosis rate of PCa but did not reduce overall mortality or disease-specific mortality.^[14]^ To improve specificity and reduce unnecessary biopsy procedures, exploration of new serum markers for early detection of PCa is still needed. Our research findings indicated a significant decrease in serum ACSL4 levels in PCa patients and its potential as an adjunct for early PCa diagnosis.

In light of the controversy surrounding PSA screening, there has been an increasing focus on exploring alternative methods for the early diagnosis of PCa. Stefancu et al discovered that combining surface-enhanced Raman scattering spectroscopy of serum samples with serum PSA levels can enhance the accuracy of PCa diagnosis in patients.^[15]^ A meta-analysis by Song et al indicated that miRNAs are suitable for predicting different stages of PCa, and detecting miRNAs is an effective approach for controlling patient prognosis and evaluating treatment efficacy.^[16]^ Additionally, a study by Li et al revealed that levels of serum eprinA2 and exosomal eprinA2 were significantly elevated in PCa patients compared to those with BPH, and exosomal eprinA2 demonstrated superior diagnostic efficiency in differentiating PCa patients from BPH patients compared to serum eprinA2 and PSA.^[17]^ In our study, ROC curve analysis showing the ability of serum ACSL4 to discriminate between PCa and BPH indicates its potential as a diagnostic tool. Our findings suggested that serum ACSL4 may serve as a complementary or alternative marker to PSA in PCa screening. ROC curves also suggested that combined ACSL4 and PSA might have better diagnostic outcomes.

In clinical practice, multiple studies have focused on the role of ACSL4 in patient diagnosis and prognosis. In cholangiocarcinoma, the infiltration level of immune cells is associated with ACSL4 levels, making ACSL4 a potential novel biomarker for cholangiocarcinoma.^[18]^ In patients with pulmonary nodules, serum ACSL4 levels are significantly lower in malignant nodule patients compared to those with benign nodules.^[19]^ In the treatment of hepatocellular carcinoma, ACSL4 is crucial for sorafenib-induced ferroptosis and can be used to predict hepatocellular carcinoma sensitivity to sorafenib.^[20]^ However, no studies have reported the clinical significance of serum ACSL4 in PCa patients. Our study results indicated significantly lower serum ACSL4 levels in PCa patients compared to those with BPH. The correlation between serum ACSL4 and PSA levels also suggested that ACSL4 could complement PSA as a diagnostic tool. Additionally, in animal studies, Wang et al found that induced ferroptosis in vivo could inhibit PCa tumor growth and metastasis, improving mouse survival rates.^[12]^ Modulating ACSL4 levels could promote docetaxel resistance in PCa.^[21]^ These findings collectively indicated that ACSL4 plays an important role in the progression, treatment, and prognosis of PCa. Our study validated these findings, as K–M analysis showed lower survival rates and higher recurrence rates in PCa patients with lower serum ACSL4 levels, suggesting that ACSL4 may serve as a prognostic indicator.

There are certain limitations to our study that should be acknowledged. Firstly, the small sample size and the lack of consideration for factors such as lifestyle and genetic predispositions of both PCa and BPH patients in the analysis are notable limitations. These confounding factors may introduce bias and affect the reliability of the study results. Secondly, our analysis only assessed a limited number of serum biomarkers, which may have excluded other potentially relevant variables. Furthermore, it is worth noting that the mortality rate among PCa patients is generally low. However, in our study, we observed 5 deaths during the follow-up period. Conducting survival analysis on these patients may further influence the generalizability of our findings. Lastly, further in-depth research is needed to elucidate the molecular mechanisms by which ACSL4 is involved in the development of PCa. Future investigations should focus on larger patient cohorts, longitudinal follow-up, and the integration of ACSL4 with other clinical parameters to determine its potential as a standalone or combined marker for early diagnosis and prognosis assessment in PCa.

## 5. Conclusion

Our findings indicated that the serum levels of ACSL4 were significantly decreased in PCa patients compared to BPH patients. Moreover, ACSL4 was identified as a risk factor associated with PCa patients. The identification of serum ACSL4 as a potential biomarker for early PCa diagnosis and prognosis assessment opens up new avenues for improving patient management and treatment decision-making.

## Author contributions

**Conceptualization:** Hao Wang.

**Data curation:** Hao Wang, Hanfeng Xu.

**Formal analysis:** Hao Wang, Hanfeng Xu.

**Funding acquisition:** Yuan Yang.

**Methodology:** Hanfeng Xu.

**Writing – original draft:** Hao Wang.

**Writing – review & editing:** Yuan Yang.
